# Biophysical and chemical stability of surfactant/budesonide and the pulmonary distribution following intra-tracheal administration

**DOI:** 10.1080/10717544.2019.1618418

**Published:** 2019-06-17

**Authors:** Chung-Ming Chen, Chien-Hsiang Chang, Chih-Hua Chao, Mei-Hui Wang, Tsu-Fu Yeh

**Affiliations:** a Department of Pediatrics, School of Medicine, College of Medicine, Taipei Medical University, Taipei, Taiwan;; b Department of Pediatrics, Taipei Medical University Hospital, Taipei, Taiwan;; c Maternal Child Health Research Center, College of Medicine, Taipei Medical University, Taipei, Taiwan;; d Department of Chemical Engineering, National Cheng Kung University, Tainan, Taiwan;; e Department of Pharmacy, China Medical University, Taichung, Taiwan;; f Institute of Nuclear Energy Research, Taoyuan, Taiwan;; g Department of Pediatrics, Children’s Hospital, China Medical University, Taichung, Taiwan

**Keywords:** Bronchopulmonary dysplasia, surface activity, budesonide, respiratory distress syndrome

## Abstract

Intra-tracheal instillation of budesonide using surfactant as a vehicle significantly decreased the incidence of bronchopulmonary dysplasia or death in preterm infants. The formularity of surfactant supplemented with budesonide and biophysical and chemical stability of the suspension has not been well reported. The aims are to investigate the biophysical and chemical stability of two surfactant preparations, Survanta and Curosurf, supplemented with budesonide. Biophysical property of the surface tension of Survanta and Survanta/budesonide suspension and of Curosurf and Curosurf/budesonide suspension was conducted by a pulsating bubble surfactometer and by a drop shape tensiometer. Chemical stability of Survanta/budesonide and of Curosurf/budesonide suspensions was tested by high-performance liquid chromatography analysis (HPLC). Pulmonary distribution of Survanta/^18^F-budesonide suspension was examined by a Nano/PET digital scan in rats. The Marangoni effect of Survanta, Curosurf, and budesonide was tested by digital high speed photography. For Survanta supplemented with budesonide, with a concentration ratio of ≥50, the surface tension-lowering activity was minimally affected. Similarly, the surface tension-lowering activity of Curosurf was not significantly affected by addition of budesonide, if the concentration ratio was ≥160. With these concentration ratios of both suspensions, HPLC analysis revealed no new compounds identified. Curosurf as compared to Survanta exhibited a significantly higher Marangoni effect. We conclude that with current dosage recommended for Survanta and Curosurf, both surfactant/budesonide suspensions are biophysically and chemically stable. Both surfactants can act as an effective vehicle for budesonide delivery.

## Introduction

Respiratory distress syndrome (RDS) is a major cause of morbidity and mortality in preterm neonates (Sakonidou & Dhaliwal, 2015). Despite recent improvements in the management of RDS in preterm infants, bronchopulmonary dysplasia (BPD) remains a major cause of morbidity and mortality during the first year of life, and many infants experience significant respiratory morbidity throughout childhood (Jacob et al., 1998). Some abnormal lung functions may persist into adulthood (Wong et al., 2008). However, no effective therapy is currently clinically available to prevent the development and long-term pulmonary sequelae of BPD.

The pathogenesis of BPD is multifactorial, and pulmonary inflammation is considered to play the central role for the development of BPD. Inhaled and systemic corticosteroids have been used to treat or prevent BPD (Cole et al., 1999; Shah et al., 2012a,b). However, systemic corticosteroid is not generally recommended because of long-term adverse effects (American Academy of Pediatrics, Committee on Fetus and Newborn, 2002, 2010). Administering inhaled glucocorticoids to preterm infants is technically challenging and the effects are limited. Therefore, it is important to find a therapeutic method that reduces the systemic adverse events of corticosteroids while at the same time retaining local anti-inflammatory effects on the lungs. Budesonide is a glucocorticoid with strong local anti-inflammatory effects. A pilot study showed that intra-tracheal instillation of budesonide, using surfactant as a vehicle, significantly improved pulmonary status (Yeh et al., 2008). A multi-center, randomized clinical trial using intra-tracheal administration of Survanta/budesonide significantly decreased the incidence of BPD or death without immediate adverse effect compared with Survanta alone (Yeh et al., 2016).

Animal-derived surfactant extracts seem to be the more desirable choice when compared to synthetic surfactants in the treatment and prevention of RDS (Ardell et al., 2015). The most common clinically used animal derived surfactants are Survanta or Curosurf. A systematic meta-analysis revealed that high dose Curosurf exhibits superior short-term clinical outcomes when compared with Survanta in the treatment of preterm infants with established RDS (Singh et al., 2011). The biophysical and chemical stability of Survanta/budesonide have been partially presented as supplement to the original paper, but have not been fully reported on Curosurf/budesonide (Yeh et al., 2016). This paper will report in detail the biophysical and chemical stability of either Survanta/budesonide or Curosurf/budesonide mixture. We will also evaluate *in vivo* the original paper, how surfactant influences on the pulmonary distribution of radio-isotope labeled budesonide and the in vitro Marangoni effect of Survanta, Curosurf, and budesonide.

## Methods

### Surfactant preparations

Survanta (Abbott Ltd, Columbus, OH, USA) was prepared by lipid extraction of minced bovine lungs and contains approximately 84% phospholipids, 1% hydrophobic surfactant proteins proteins (SP-B and SP-C), and 6% free fatty acids. The solution was supplied at a concentration of 25 mg/ml of phospholipid suspended in 0.9% sodium chloride solution. Curosurf (Chiesi Farmaceutici S.p.A., Parma, Italy) was produced from minced pig lungs and consists of 99% phospholipids and 1% hydrophobic surfactant proteins. The solution was supplied at a concentration of 80 mg/ml of phospholipid fraction.

### Sample preparation

Both surfactants and budesonide (Pulmicort nebulizing suspension, Astra Zeneca, London, UK, each ml of the suspension contains 0.5 mg budesonide) were studied at a wide range of concentration ratios with respect to the phospholipids in Survanta and Curosurf. Budesonide was added at a Survanta/budesonide concentration ratio of 25:1, 50:1, and 100:1 and at a Curosurf/budesonide concentration ratio of 160:1.

### *In vitro* biophysical and chemical stability of surfactant supplemented with budesonide

The surface tension data were determined by commercial pulsating bubble tensiometer and drop shape tensiometer (model FTA-1000 B class, First Ten Angstroms, USA), respectively. For the surface tension measurements obtained by the commercial pulsating bubble tensiometer (Enhorning, 1977), a bubble was created in the aqueous sample and the pressure difference across the air/liquid interface, used to evaluate the surface tension at the interface, was followed with time. The equilibrium surface tension was then determined until no significant change was detected in the surface tension observations (Chang & Franses, 1994). As for the commercial drop shape tensiometer, a drop of samples was formed in the air environment and the surface tension at the interface of the drop was then evaluated from the profile of the drop (Zuo & Neumann, 2005). Although the drop profile, or corresponding surface tension, could be followed with time, evaporation of the drop could not be totally avoided (the drop size would be decreased with time) and it is unfeasible to measure the surface tension for a very long time. Depending on the dynamic adsorption behavior of the surface active components in the samples, the measured surface tension may not be close to the equilibrium surface tension. For Curosurf suspensions, the dynamic surface tension was decreased fast. However, the adsorption timescale of budesonide suspension was long, or the dynamic surface tension was decreased very slowly. In order to fairly compare the surface tension data of the samples to explore the added effect of budesonide suspension, the data reported in Table 2 were obtained right after the sample drops were formed.

To test the chemical stability of the Survanta supplemented with budesonide, high performance liquid chromatography (HPLC) analysis were performed at 0, 1, 4, 8, 12, and 24 hours after Survanta/budesonide suspension with a concentration ratio of 25:1, 50:1, and 100:1. To test the chemical stability of the Curosurf supplemented with budesonide, HPLC analyses were performed for 0, 8, 12, 24, and 48 hours with the Curosurf/budesonide concentration ratio of 160:1, the lowest Curosurf/budesonide concentration that will maintain normal Curosurf biophysical activity.

### *In vivo* pulmonary distribution of Survanta supplemented with budesonide

Male Sprague-Dawley rats (200–250 g) were maintained in a pathogen-free facility. Animals were kept at approximately 25 °C and pelleted food and water were available *ad libitum* throughout the experiment. The study protocol was approved by the Institute of Nuclear Energy Research.

#### Preparation of ^18 ^F-Budesonide

Radiosynthesis was done by using a commercial apparatus (TRACERlab FX F-N; GE Healthcare, USA), we dried aqueous ^18 ^F-fluoride ion (∼10 mCi) by iterative cycles of addition and evaporation of acetonitrile, followed by complexation with K+-K2.2.2/K_2_CO_3_. The complex was then reacted with budesonide (10 mg) at 110 °C for 10 min in anhydrous dimethyl fluoride (0.6 ml), passed through a Sep-Pak C-18 to remove the free ^18 ^F ion and followed by hydrolysis with NaOMe/methanol (1.0 ml). The specific radioactivity is 30 µCi/mg or greater. The radiolabelled products were analyzed with radio-HPLC and radio-thin-layer chromatography (radio-TLC). The results of radio-HPLC method were used for comparison and confirmation of that of radio-TLC. The radiochemical purity, determined by thin-layer chromatography, needs to be greater than 98%.

#### Intratracheal administration

Rats were anesthetized with 1.5% isoflurane and then positioned against an angled restraining stand and exposed to locate the trachea. The radiolabelled solutions were prepared and mixed immediately prior to intratracheal instillation. Survanta and budesonide (Astra Zeneca, Lund, Sweden) were given intratracheally at dosages of 12.5 mg/kg and 0.25 mg/kg, respectively. The rats were randomly assigned to two treatment groups (*n* = 3 per group) as follows: 0.2 mCi of ^18 ^F-budesonide and 0.2 mCi of surfactant/^18^F-budesonide (mix by the weight ratio of 1 to 50).

##### Measurement of radioactivity and monitoring of pulmonary distribution

The radioactivity was measured by Capintec dose calibrator. The specific radioactivity was measured by radio-HPLC and the radioactivity in lung was expressed as percentage injected dose (%ID).

### *In vitro* comparison of Marangoni effect between Survanta and Curosurf

To assess the Marangoni effect, the movement of a paper sheet at the air/water interface after a drop of sample was introduced and monitored by a high-speed camera (high-speed photography) and the speed of the paper sheet movement was estimated from distance/time. The following samples: SDS (sodium dodecyl sulfate, a commercial surfactant), budesonide, Survanta, and Curosurf were assessed for the Marangoni effect. Each drop of sample was estimated to be about 0.002 ml, each sample was tested for four times, and the average speed of the paper sheet movement was calculated.

### Statistical analysis

Data are expressed as means ± SD. Between-group comparisons were made using Student’s *t*-test. Differences were considered significant at *p* < .05.

## Results

### Biophysical and chemical stability of Survanta supplemented with budesonide

When a Survanta suspension was supplemented with budesonide suspension, with a concentration ratio of 12.50:0.25 mg/ml (50:1) or greater, the dynamic surface activity of Survanta suspension was minimally affected (Table 1). Based on these results, we decided that the dosage between Survanta/budesonide ratios for neonates should be ≥50:1.

**Table 1.** Surface tension behavior of Survanta, budesonide, and their mixtures.

System	Phospholipid concentration (mg/ml)	Budesonide concentration (mg/ml)	γ_e_	γ_min_(mN/m)	γ_max_
Surfactant	25.0	0	19	0	46
	12.5	0	21	0	51
	1.0	0	21	5	49
Budesonide	0	0.5	31	27	47
	0	0.25	33	29	49
Surfactant/	12.5	0.25	20	0	41
budesonide	1.0	0.25	28	20	45

mN/m: milli-Newton/meter; γ_e_: equilibrium surface tension; γ_min_: minimum surface tension obtained at a pulsation rate of 20 cycles/min; γ_max_: maximum surface tension obtained at a pulsation rate of 20 cycles/min.

**Figure 1.**
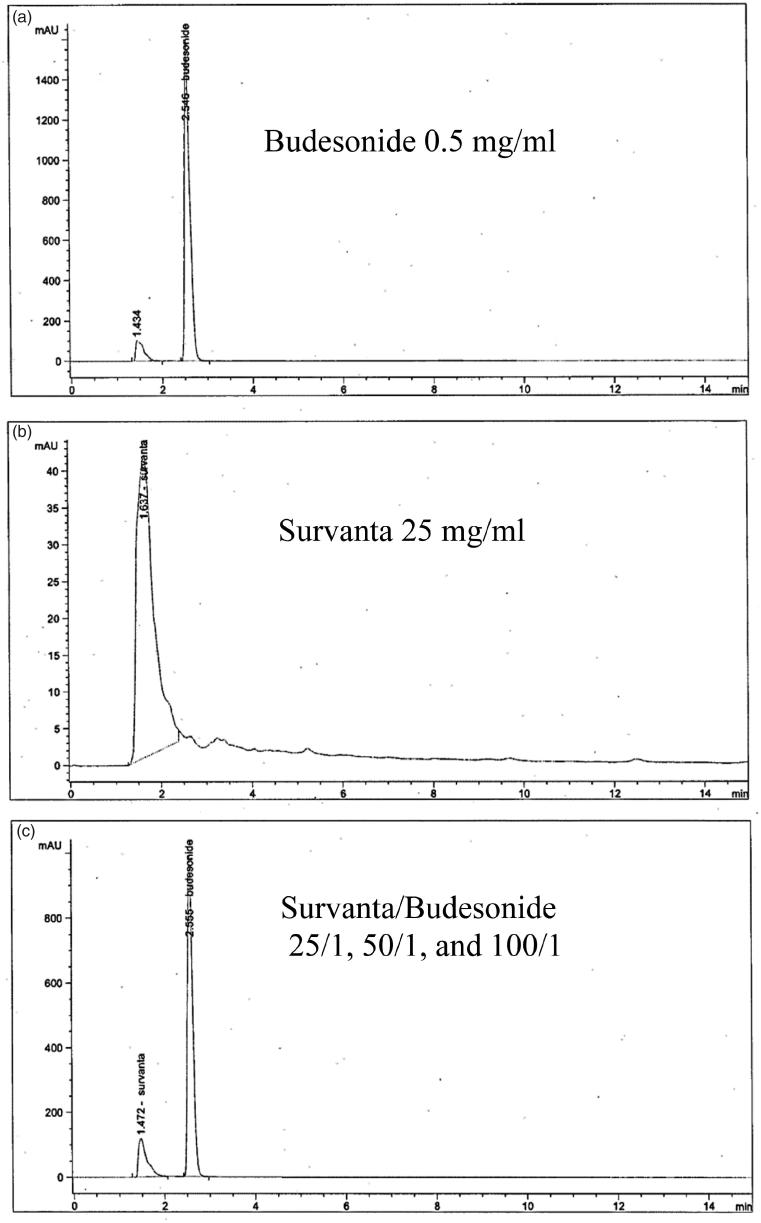
Representative HPLC chromatograms of (a) budesonide, (b) Survanta, and (c) Survanta/budesonide mixture, comprising different concentration ratio of mixture (25:1, 50:1, and 100:1) at 0, 1, 4, 8, 12, 24 h after mixing of the two drugs. There was no new compound identified during these tests, indicating that Survanta/budesonide mixture is chemically stable.

The HPLC analysis revealed that no new compounds were identified at 0, 1, 4, 8, 12, and 24 hours after mixing Survanta and budesonide at various concentration ratios (Figure 1(a–c)). Thus, the mixture of budesonide and Survanta appears to be chemically stable.

### Biophysical and chemical stability of Curosurf supplemented with budesonide

**Figure 2.**
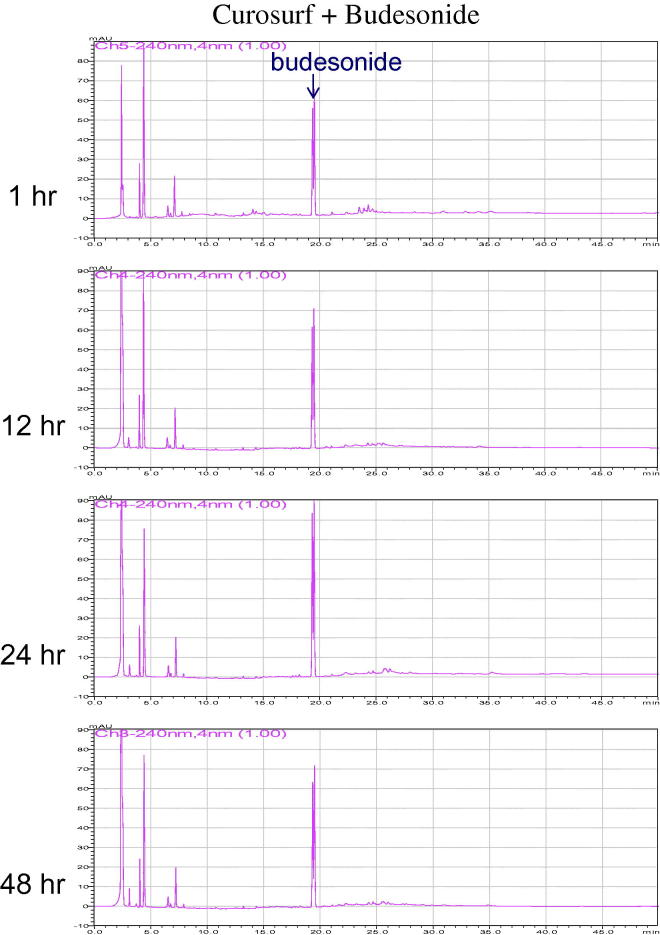
Representative HPLC chromatograms of Curosurf/budesonide suspension at 1, 12, 24, and 48 hours after mixing of the two drugs. There was no new compound identified during these tests (arrow denotes budesonide), indicating that Curosurf/budesonide suspension is chemically stable.

When Curosurf was supplemented with budesonide with a concentration ratio of ≥ 160 or greater, the surface tension of Curosurf was not significantly affected (Table 2). The HPLC analysis of Curosurf supplemented with budesonide, no new compound can be identified for 48 hours (Figure 2).

**Table 2.** Surface tension behavior of Curosurf, budesonide, and their mixtures.

System	Phospholipid concentration (mg/ml)	Budesonide concentration (mg/ml)	γ (mN/m)
Curosurf	80.0	0	27.0 ± 0.7 (*n* = 8)
	40.0	0	27.4 ± 0.7 (*n* = 7)
	1.0	0	29.6 ± 0.6 (*n* = 6)
Budesonide	0	0.5	49.8 ± 0.6 (*n* = 6)
	0	0.25	53.2 ± 0.3 (*n* = 6)
Curosurf/	40.0	0.25	28.4 ± 0.5 (*n* = 6)
Budesonide	1.0	0.25	34.4 ± 0.8 (*n* = 4)

The surface tension data were obtained right after the drops were formed.

mN/m: milli-Newton/meter; γ: surface tension.

### Comparison of Marangoni effect between Survanta and Curosurf

The Marangoni effect was expressed as speed movement of paper sheet following a drop of SDS: 8.509 ± 1.067 cm/sec, budesonide: 0.990 ± 0.131 cm/sec, Survanta: 2.755 ± 0.61 cm/sec, and Curosurf: 11.277 ± 2.10 cm/sec. The difference in speed movement between Survanta and Curosurf is statistically significant (*p* < .001).

### *In vivo* pulmonary distribution of Survanta supplemented with budesonide in rats

**Figure 3.**
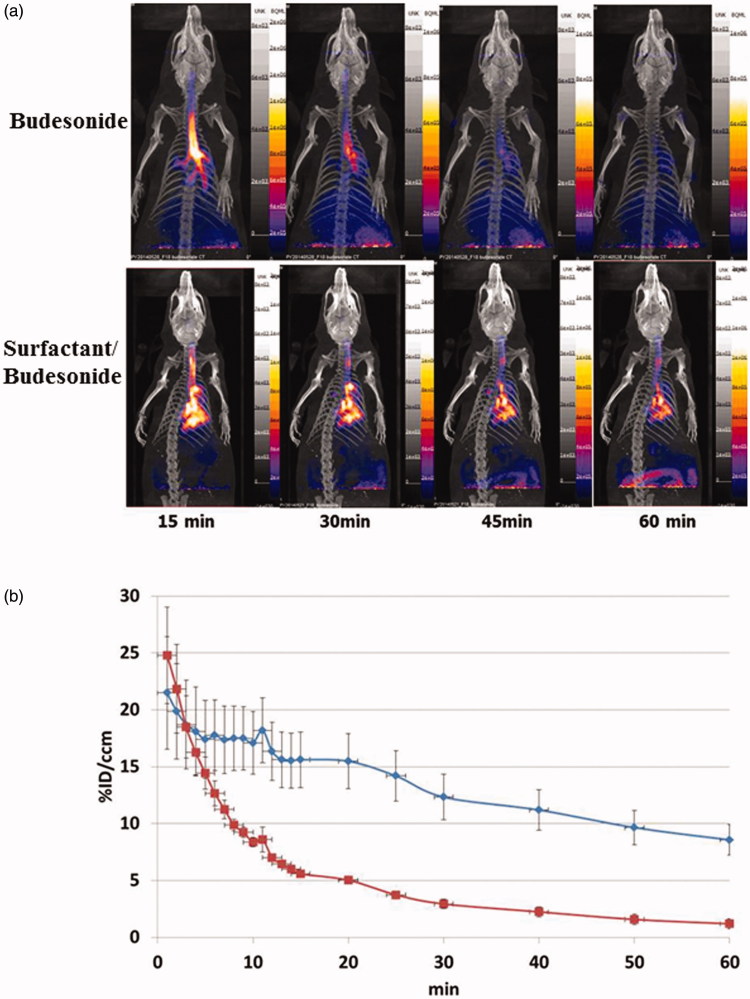
The ^18 ^F-budesonide bio-distribution (a) and radioactivity (b) in the Sprague–Dawley rats intratracheally injected with surfactant/^18^F-budesonide (*n* = 3, blue diamond) or ^18 ^F-budesonide alone (*n* = 3, red square). The ^18 ^F-budesonide was distributed more into the peripheral lungs and the accumulated ^18 ^F-budesonide radioactivity was higher in the rats supplemented with surfactant than in the rats without surfactant during the study period.

The radioactivity of ^18 ^F-budesonide was most strongly detected near the trachea at 15 min after intra-tracheal injection (Figure 3(a)). Almost no radioactivity was seen in the lung region of the rats injected with ^18 ^F-budesonide alone at 60 min. The radioactivity of ^18 ^F-budesonide was distributed more in the peripheral lung and stayed longer in rats supplemented with surfactant than in the rats without surfactant. Rats intratracheally injected with surfactant/^18^F-budesonide mixture exhibited an approximately 200% increase in radioactivity compared with rats that received ^18 ^F-budesonide alone during the study period (Figure 3(b)).

## Discussion

Intratracheal administration of Survanta/budesonide suspension has been shown to decrease the incidence of BPD or death without immediate adverse effect in very-low-birth-weight infants with severe RDS. In this study, we found that Survanta supplemented with budesonide with concentration ratio ≥50 or Curosurf supplemented with budesonide with a ratio of ≥160, did not significantly affect the surface tension reducing property of Survanta or Curosurf. With these concentration ratios, HPLC revealed chemical stability in both suspensions at least for 24-48 hours. Nano/PET digital scans showed that Survanta facilitated the distribution of ^18 ^F-budesonide into the lung. Curosurf exhibited a significantly higher Marangoni effect as compared to Survanta, therefore Curosurf will more likely facilitate the distribution much fast than that of Survanta.

Corticosteroids may alleviate pulmonary inflammation. However, systemic corticosteroids are known to cause serious adverse effects. If the local anti-inflammatory effect on the lung can be used while the systemic side effects are curtailed, corticosteroids can be extremely useful in preventing BPD. Previous studies showed that intratracheal instillation of budesonide, using surfactant as a vehicle; significantly reduce the incidence of BPD or death (Yeh et al., 2008, 2016). However, the mechanism that mediates these beneficial effects is not yet well understood. This study provides in vitro and in vivo evidences that surfactant supplemented with budesonide was biophysically and chemically stable and Curosurf facilitated the in vivo pulmonary distribution of budesonide.

Budesonide suspension is a sterile suspension that contains the active ingredient budesonide, and the inactive ingredients citric acid, edetate disodium dihydrate, polysorbate 80, sodium chloride, sodium citrate, and water. A water-insoluble suspension of budesonide was sterically stabilized using polysorbate 80 (Owen et al., 2009). Polysorbate 80 is a nonionic surfactant and emulsifier derived from polyethoxylated sorbitan and oleic acid. Disodium edetate is a parenteral chelating agent. Polysorbate 80 and disodium edetate have the ability to reduce surface tension (Cain et al., 2007). These factors may not influence the results as we used only one brand of budesonide in this study.

Animal derived surfactant have been shown to have several advantages over the synthetic surfactants and are the most commonly used surfactant preparations in the treatment and prevention of RDS (Ardell et al., 2015). The most common clinically used animal-derived surfactants are either extracted from minced cow lung with the addition of DPPC, palmitic acid and tripalmitin (Survanta) or extracted from minced pig lung (Curosurf). The studied natural lung surfactant replacements are used as a bolus instilled into a baby’s lung. The surfactant spreads throughout the lung in a very short time assisted by mechanical ventilation and probably Marangoni flow (Cassidy et al., 2001; Halpern et al., 2008). The Marangoni effect is the mass transfer along an interface between two fluids due to surface tension gradient and surface area changes during breathing cycle (Sosnowski, 2018).

Imaging techniques have been used to study the delivery of intratracheally instilled medications. Nano/PET digital scans of ^18 ^F-budesonide deposition provide exact localization of deposited dose within the 3-dimensional anatomy of the lung. These images provide both a qualitative picture of ^18 ^F-budesonide deposition in the lungs and a means to quantify the dose. In this study, we demonstrated Survanta enhanced ^18 ^F-budesonide distribution into the peripheral lung. Surface tension gradient-driven Marangoni flow can move either surfactant dispersions or drug-carrying preparations through the lung. Koch et al demonstrated Marangoni flows driven by soluble surfactants may enhance drug delivery to diseased lungs (Koch et al., 2011). In this study, we found that Curosurf exhibited a significantly higher Marangoni effect compared to that of Survanta. These results were consistent with the findings of Hermans et al who observed Survanta exhibits pronounced aggregate structures and a higher surface viscoelasticity that that of Curosurf (Hermans et al., 2015). These spreading behaviors indicate that Curosurf may achieve a faster speed for budesonide delivery into the lungs.

In conclusion, we found with current dosage recommended for RDS, Survanta or Curosurf are effective vehicles to facilitate the delivery of budesonide into the lungs. Curosurf exhibited an enhanced in vitro Marangoni effect compared to Survanta.
